# Acute Kidney Injury in Critically Ill Children: A Retrospective Single-Center Study

**DOI:** 10.3390/children13070925

**Published:** 2026-07-14

**Authors:** Samah Al-Harbi, Fatmah Saad Waggass, Nadeem Shafique Butt

**Affiliations:** 1 Department of Pediatrics, Faculty of Medicine, King Abdulaziz University, Jeddah 22252, Saudi Arabia; smhalharbi@kau.edu.sa; 2 Department of Pediatrics, King Abdulaziz University Hospital, Jeddah 22252, Saudi Arabia; 3 Department of Pediatrics, King Fahd Armed Forces Hospital, Jeddah 21159, Saudi Arabia; fatmahwaggass@gmail.com; 4 Department of Family and Community Medicine, King Abdulaziz University, Rabigh 25732, Saudi Arabia

**Keywords:** acute kidney injury, pediatric intensive care unit, KDIGO criteria, mortality, renal replacement therapy, critical care

## Abstract

**Highlights:**

**What are the main findings?**

- Acute kidney injury (AKI) occurred in 13.5% of eligible critically ill children admitted to a tertiary PICU. Stage 3 AKI was associated with the highest mortality rate (27.8%), and younger age and a greater number of clinical complications were associated with more severe AKI.
- Inotropic support (adjusted OR 8.51, 95% CI: 2.44–29.63; *p* < 0.001), number of clinical complications (adjusted OR 4.38, 95% CI: 2.28–8.42; *p* < 0.001), and PRISM IV score (adjusted OR 1.07, 95% CI: 1.00–1.14; *p* = 0.042) were associated with PICU mortality in the multivariable model. These findings support the clinical relevance of hemodynamic instability, multiorgan complication burden, and baseline illness severity. Gender was not significant in the multivariable model (adjusted OR 2.24; *p* = 0.212).

**What are the implications of the main findings?**

- Renal replacement therapy was used in only 4.3% of the cohort despite substantial Stage 3 AKI severity, suggesting a potential care gap and the need for standardized nephrology referral protocols and clear RRT initiation thresholds in resource-limited PICU settings.
- Early AKI recognition using KDIGO criteria, closer monitoring of younger children, and prompt hemodynamic stabilization may help reduce morbidity and mortality in critically ill pediatric patients.

**Abstract:**

Background and Objective: Acute kidney injury (AKI) is an important contributor to morbidity and mortality in critically ill children. This study aimed to determine the incidence, clinical characteristics, and outcomes of AKI in a tertiary pediatric intensive care unit (PICU). Methods: We conducted a retrospective observational cohort study at King Abdulaziz University Hospital, Jeddah, Saudi Arabia, from January 2018 to December 2022. Children aged 29 days to 14 years who were admitted to the PICU for ≥48 h were included. AKI was defined and staged according to the 2012 KDIGO criteria. Neonates, patients with chronic kidney disease, elective or post-operative admissions, and patients with do-not-resuscitate orders were excluded. Results: Of 1200 eligible admissions, 162 (13.5%) developed AKI: Stage 1, 44 (27.2%); Stage 2, 64 (39.5%); and Stage 3, 54 (33.3%). Greater AKI severity was associated with younger age, greater inotropic requirement, and longer mechanical ventilation (all *p* < 0.05). Renal replacement therapy was used in 4.3% of the cohort. Overall PICU mortality was 16.7% and increased to 27.8% in Stage 3 AKI (*p* = 0.006). Inotropic support (adjusted OR 8.51; *p* < 0.001), number of complications (adjusted OR 4.38; *p* < 0.001), and PRISM IV score (adjusted OR 1.07; *p* = 0.042) were associated with mortality in the multivariable model; gender was not significant (adjusted OR 2.24; *p* = 0.212). Conclusions: AKI severity was associated with worse clinical outcomes. Early recognition, hemodynamic stabilization, timely nephrology involvement, and post-AKI follow-up are important components of care.

## 1. Introduction

Acute kidney injury (AKI) is an important and increasingly recognized condition in pediatric intensive care units (PICUs), where it is associated with increased morbidity, prolonged mechanical ventilation, longer hospital stay, and higher mortality [1,2,3]. The use of serum creatinine as a key marker of renal function has shaped the clinical understanding of AKI, and its implications in critically ill children became increasingly clear in the late 1990s and early 2000s [4]. Standardized definitions, including the pediatric Risk, Injury, Failure, Loss of kidney function, and End-stage renal disease (pRIFLE) criteria and the Kidney Disease: Improving Global Outcomes (KDIGO) guidelines, have improved AKI diagnosis, staging, and epidemiological comparison [5,6,7]. These criteria allow clinicians and researchers to classify the severity of renal impairment and compare outcomes across institutions and regions.

The reported incidence of AKI varies widely according to the population studied, diagnostic criteria used, and healthcare setting. In pediatric patients, AKI has been reported in approximately 5% to 25% of PICU admissions, with higher rates in subgroups such as children with severe sepsis, hematology–oncology conditions, or cardiac surgery [8,9,10]. The pathophysiology of AKI in critically ill children is multifactorial and may involve ischemic, nephrotoxic, and inflammatory injury to the renal parenchyma. AKI is often accompanied by volume overload, metabolic acidosis, and electrolyte disturbances, including hyperkalemia, hypocalcemia, and hyperphosphatemia. Although these abnormalities are linked to worse clinical trajectories, their precise relationship with mortality remains an area of active investigation [11,12]. The systemic consequences of AKI, often described as organ cross-talk, may further complicate the clinical course because renal dysfunction can worsen lung injury, impair cardiac function, and dysregulate immune responses.

The consequences of AKI extend beyond the period of acute renal impairment. In children, AKI is associated with longer mechanical ventilation and prolonged PICU stay, which underscores its effect on overall recovery [13,14]. Mechanical ventilation is particularly relevant because AKI-related fluid overload can contribute to pulmonary edema, reduced lung compliance, and delayed ventilator weaning. In addition, children who experience AKI have a higher risk of death than those without kidney injury, and survivors may be at increased risk of chronic kidney disease (CKD) later in life [15,16]. Despite this growing recognition, AKI remains underdiagnosed and undertreated in many resource-limited settings, highlighting the need for region-specific epidemiological data.

This study aimed to determine the incidence, clinical characteristics, risk factors, and outcomes of AKI in critically ill children admitted to a tertiary PICU in Saudi Arabia using standardized KDIGO criteria. The findings are intended to inform local management protocols and contribute to regional data on pediatric AKI. Contemporary AKI frameworks increasingly view AKI as part of a continuum that may progress to acute kidney disease (AKD) and chronic kidney disease (CKD), making post-AKI surveillance clinically important even after apparent recovery from the acute PICU episode. This study adds to the literature by providing five-year epidemiological data from a Saudi Arabian tertiary PICU, describing a regional comorbidity pattern dominated by hematology–oncology conditions, examining the relationship between hemodynamic instability, complication burden, and mortality, and documenting low RRT use despite a substantial proportion of Stage 3 AKI.

## 2. Materials and Methods

### 2.1. Study Design and Setting

This retrospective observational cohort study was conducted in the Pediatric Intensive Care Unit of King Abdulaziz University Hospital, a tertiary academic medical center in Jeddah, Saudi Arabia. The study period extended from 1 January 2018, to 31 December 2022. The December 2022 cutoff reflects the locked retrospective dataset used for IRB-approved data extraction, cleaning, statistical analysis, and manuscript preparation. Ethical approval was obtained from the Institutional Review Board of King Abdulaziz University Hospital before data collection. Because the study was retrospective and observational, the requirement for individual informed consent was waived.

### 2.2. Patient Selection

All patients aged 29 days to 14 years who were admitted to the PICU for at least 48 h were eligible for inclusion. Patients were excluded if they were 28 days of age or younger, had pre-existing chronic kidney disease (CKD) or end-stage renal disease (ESRD), were admitted electively or post-operatively, or had do-not-resuscitate (DNR) status at admission. Patients with insufficient data to determine AKI status were also excluded. This category included patients without a documented or estimable baseline creatinine, patients with missing key urine output documentation, and patients with incomplete admission records that precluded KDIGO staging.

### 2.3. AKI Definition and Staging

AKI was defined and staged according to the 2012 KDIGO Clinical Practice Guidelines, using serum creatinine criteria and/or urine output (UOP) criteria, whichever produced the higher stage for each patient. Baseline serum creatinine was defined as the lowest creatinine value documented within three months before PICU admission. For patients without previous creatinine measurements, baseline creatinine was estimated using the Schwartz formula [17] based on age-specific normal glomerular filtration rate (GFR) values, a standard approach in pediatric retrospective AKI research. In the analysis dataset, admission urine output, initial creatinine, and highest creatinine were available for all 162 AKI patients, and final KDIGO grades were Stage 1 (n = 44), Stage 2 (n = 64), and Stage 3 (n = 54). However, the dataset did not include separate audit fields indicating whether the final KDIGO stage was triggered by creatinine-only criteria, urine-output-only criteria, or both criteria, and it did not distinguish measured baseline creatinine from Schwartz-estimated baseline creatinine. Therefore, criterion-source and baseline-source counts could not be reconstructed reliably from the available analysis file. The KDIGO stages were defined as follows.

Stage 1: Serum creatinine increase of ≥0.3 mg/dL (26.5 µmol/L) within 48 h, or 1.5–1.9× baseline; and/or UOP < 0.5 mL/kg/h for 6–12 h.

Stage 2: Serum creatinine 2.0–2.9× baseline; and/or UOP < 0.5 mL/kg/h for >12 h.

Stage 3: Serum creatinine ≥3.0× baseline, or absolute serum creatinine ≥ 4.0 mg/dL (353.6 µmol/L), or initiation of RRT, or estimated GFR < 35 mL/min/1.73 m^2^ (in patients < 18 years); and/or UOP < 0.3 mL/kg/h for ≥24 h, or anuria for ≥12 h.

### 2.4. Data Collection

Data were extracted from electronic medical records and the PICU nursing documentation system. Collected variables included demographic information (age, sex, weight, and height), admission source, primary diagnosis, underlying comorbidities, Pediatric Risk of Mortality (PRISM IV) score at admission, inotropic and respiratory support, duration of mechanical ventilation, ventilator-free days (VFDs), peak serum creatinine, estimated GFR, UOP at PICU admission, nephrotoxic medication exposure (e.g., vancomycin, acyclovir, and amikacin), diuretic use (furosemide), RRT use, number of clinical complications, PICU length of stay, and final health outcome (survival without new morbidity, survival with new morbidity, or death). Institutional criteria for initiating RRT generally included refractory fluid overload (typically >10–15% above baseline body weight), severe metabolic acidosis (pH < 7.1) unresponsive to medical therapy, symptomatic uremia, or refractory hyperkalemia; however, final initiation decisions were made at the discretion of the consulting pediatric nephrologist. For this study, clinical complications were defined as discrete adverse events documented during the PICU stay in nursing or physician records. Counted complications included fluid overload (≥10% above baseline body weight), metabolic acidosis requiring intervention (pH < 7.2), hyperkalemia requiring active management (serum K+ > 6.0 mEq/L), hypocalcemia requiring supplementation, pulmonary edema or new respiratory deterioration attributed to fluid overload, new-onset arrhythmia, and any additional organ system failure beyond the primary presenting diagnosis documented by the treating intensivist. Each distinct event was counted separately to generate an integer per-patient count. Because several counted complications may represent consequences or severity markers of AKI rather than exposures that preceded AKI, this variable was interpreted as a marker of complication burden and illness severity, not as evidence of an independent causal pathway.

### 2.5. Statistical Analysis

Data analysis was performed using Stata statistical software (Version 16; StataCorp LLC, College Station, TX, USA) [18] and DataStatPro (Version 2.2.3) [19]. Continuous variables were assessed for normality using the Shapiro–Wilk test. Normally distributed continuous variables were presented as means with standard deviations (SD) and compared across KDIGO AKI stages using one-way Analysis of Variance (ANOVA) with post hoc Tukey correction. Non-normally distributed variables were presented as medians with interquartile ranges (IQR) and compared using the Kruskal–Wallis test. Categorical variables were presented as frequencies and percentages and compared using the Chi-square test or Fisher’s exact test, as appropriate.

Ordinal logistic regression was used to identify factors associated with AKI severity, adjusting for potential confounders including age, sex, comorbidities, inotrope requirement, duration of mechanical ventilation, number of complications, primary reason for admission, and PICU length of stay. Binary logistic regression with forward stepwise selection was initially used to identify variables associated with PICU mortality. The mortality model was then re-estimated using a comparative simultaneous-entry approach, in which all four candidate variables (gender, inotropic support, number of clinical complications, and PRISM IV score) were entered simultaneously. Each variable was also evaluated in a separate unadjusted model. The simultaneous-entry approach was used to reduce the instability associated with variable selection and to retain PRISM IV score as a pre-specified covariate for baseline illness severity. With 27 deaths and four model variables, the events-per-variable (EPV) ratio was 6.75, below the conventional threshold of 10; therefore, mortality-model estimates should be interpreted cautiously and confirmed in larger prospective studies.

## 3. Results

### 3.1. Incidence and Patient Demographics

**Figure 1:**
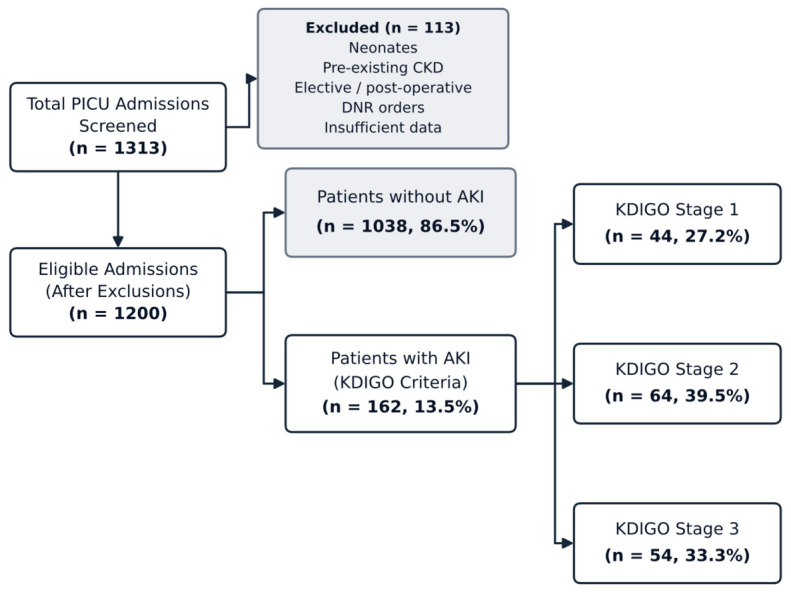
Patient enrollment and KDIGO AKI staging flow diagram.

During the five-year study period, 1313 PICU admissions were screened. After applying the predefined exclusion criteria, 1200 admissions were eligible. Of these, 162 patients developed AKI during their PICU stay, yielding an incidence of 13.5% among eligible admissions (12.3% of all screened admissions). Patient enrollment and AKI staging are shown in Figure 1.

Patients were distributed across KDIGO stages as follows: Stage 1, 44 (27.2%); Stage 2, 64 (39.5%); and Stage 3, 54 (33.3%). The median age of the AKI cohort was 3.0 years (IQR 4.5), and 53.1% were male. AKI severity was inversely associated with age; Stage 3 patients were younger (median 1 year) than Stage 1 patients (median 7 years) (*p* < 0.001). Weight and height were also significantly lower in patients with more severe AKI (*p* = 0.003 and *p* < 0.001, respectively). Underlying comorbidities were present in 59.9% of the cohort, with hematology–oncology disorders being the most common; however, the prevalence of comorbidities did not differ significantly across AKI stages (*p* = 0.237). Demographic characteristics stratified by AKI stage are presented in Table 1.

### 3.2. Clinical Characteristics and Management

Most patients were admitted from the emergency department (64.2%), and the primary reason for admission was medical in 78.4% of cases. Medical admissions were more common among patients with Stage 3 AKI (98.1%) than among those with Stage 1 AKI (61.4%) (*p* < 0.001), suggesting that severe acute medical illnesses, such as sepsis and severe respiratory failure, were major contributors to severe renal impairment in this cohort.

**Figure 2:**
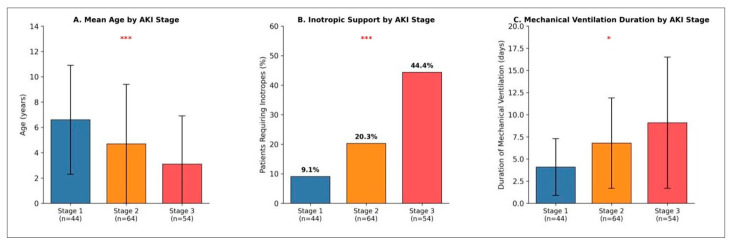
Clinical characteristics stratified by KDIGO AKI stage (Panel (**A**): age. Panel (**B**): inotropic support. Panel (**C**): mechanical ventilation duration). Note: Error bars represent standard deviations. * for *p* < 0.05; *** for *p* < 0.001.

Although PRISM IV scores were similar across AKI stages (overall mean 11.0; *p* = 0.375), clinical trajectory worsened with increasing AKI severity. Inotropic support increased from 9.1% in Stage 1 to 44.4% in Stage 3 (*p* < 0.001). Respiratory support was required in 76.5% of patients, and the duration of mechanical ventilation increased from 4.1 days in Stage 1 to 9.1 days in Stage 3 (*p* = 0.013). These clinical characteristics are shown in Figure 2.

As expected from the KDIGO definition, Stage 3 patients had the highest serum creatinine levels and the lowest estimated GFR values (*p* < 0.001). RRT was used in 4.3% of the overall cohort and was concentrated among Stage 3 patients (11.1%; *p* = 0.011). Pediatric nephrology consultation occurred in 30.2% of all cases and increased significantly with AKI severity (55.6% in Stage 3; *p* < 0.001). The mean number of clinical complications also increased with AKI severity, from 1.4 in Stage 1 to 2.3 in Stage 3 (*p* < 0.001).

### 3.3. Clinical Outcomes

**Figure 3:**
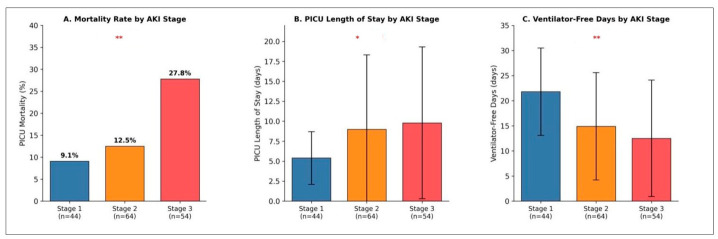
Clinical outcomes stratified by KDIGO AKI stage (Panel (**A**): mortality rate. Panel (**B**): PICU length of stay. Panel (**C**): ventilator-free days). Note: Error bars represent standard deviations. * for *p* < 0.05; ** for *p* < 0.01.

Severe AKI was associated with worse clinical outcomes across multiple domains. Mean PICU length of stay increased from 5.4 days in Stage 1 to 9.8 days in Stage 3 (*p* = 0.023). Ventilator-free days, a composite metric reflecting survival and liberation from mechanical ventilation, decreased from 21.8 days in Stage 1 to 12.5 days in Stage 3 (*p* = 0.003). Overall PICU mortality in the AKI cohort was 16.7% (27/162 patients). Mortality increased with AKI severity: 9.1% in Stage 1, 12.5% in Stage 2, and 27.8% in Stage 3 (*p* = 0.006). These outcome measures are summarized in Figure 3.

**Figure 4:**
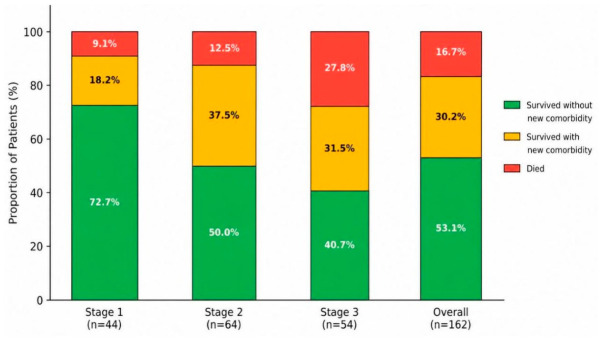
Patient outcomes by KDIGO AKI Stage.

Among survivors, the proportion of patients who survived without new comorbidities decreased from 72.7% in Stage 1 to 40.7% in Stage 3, suggesting a higher burden of morbidity among patients with severe AKI (Figure 4).

### 3.4. Predictors of AKI Severity and PICU Mortality

Ordinal logistic regression identified younger age as a significant negative predictor of AKI severity (OR 0.883 per year, 95% CI: 0.789–0.983; *p* = 0.025), indicating that each additional year of age was associated with an approximately 12% lower odds of being classified in a higher AKI stage. The number of clinical complications was significantly associated with higher AKI severity (OR 1.554, 95% CI: 1.019–2.417; *p* = 0.044). Surgical admissions were associated with lower AKI severity than medical admissions (OR 0.294, 95% CI: 0.107–0.786; *p* = 0.016). Inotrope requirement approached but did not reach statistical significance as a predictor of severity (OR 2.415; *p* = 0.054). Details are presented in Table 2.

For PICU mortality, comparative logistic regression analysis (Table 3) showed that inotropic support was the strongest mortality-associated variable in both the unadjusted model (OR 17.04, 95% CI: 6.19–46.90; *p* < 0.001) and the multivariable-adjusted model (OR 8.51, 95% CI: 2.44–29.63; *p* < 0.001). The number of clinical complications was also associated with mortality in both models (unadjusted OR 4.73, 95% CI: 2.75–8.12; *p* < 0.001; adjusted OR 4.38, 95% CI: 2.28–8.42; *p* < 0.001). PRISM IV score was significant in the unadjusted model (OR 1.05, 95% CI: 1.01–1.09; *p* = 0.018) and remained associated with mortality after multivariable adjustment (OR 1.07, 95% CI: 1.00–1.14; *p* = 0.042), suggesting that baseline illness severity remained clinically relevant after adjustment for hemodynamic and complication burden. Gender was not significant in either model (adjusted OR 2.24, 95% CI: 0.63–7.92; *p* = 0.212). Thus, the multivariable model highlighted the association of systemic hemodynamic instability, multiorgan complication burden, and baseline illness severity with PICU mortality.

## 4. Discussion

This retrospective study describes the burden of AKI among critically ill children admitted to a tertiary PICU in Saudi Arabia over a five-year period. AKI occurred in 13.5% of eligible admissions and showed a clear gradient with adverse outcomes, including longer PICU stay, fewer ventilator-free days, greater morbidity, and higher mortality. These findings emphasize the importance of renal surveillance in pediatric intensive care.

The AKI incidence in our cohort (13.5%) was lower than rates reported in large multicenter studies from North America and Europe. The Assessment of Worldwide Acute Kidney Epidemiology (AWARE) study, conducted across 32 PICUs, reported an AKI incidence of 26.9% using KDIGO criteria [8]. Other cohorts have reported incidences as high as 30–50% [9]. The lower incidence in our cohort may partly reflect the strict exclusion criteria, which excluded neonates and elective post-operative admissions, groups known to have high rates of transient AKI, particularly after cardiac surgery [20]. The predominance of hematology–oncology conditions (59.9%) as the main underlying comorbidity is consistent with other Middle East and North Africa (MENA) studies [16,21], but contrasts with North American cohorts in which cardiac surgery and sepsis are more prominent risk factors [20]. These regional differences support the need for context-specific AKI risk stratification and prevention strategies. Recent studies further support this interpretation. A 2023 Canadian multicenter cohort [22] reported AKI incidence rates consistent with AWARE benchmarks and identified KDIGO staging as a robust prognostic tool. A 2024 multinational analysis identified hemodynamic instability and multiorgan involvement as dominant markers of AKI-related mortality across diverse PICU settings, findings that are consistent with our multivariable results. Both studies reported substantially higher RRT use than in our cohort, suggesting that resource availability and institutional decision thresholds may influence RRT practice patterns across settings.

The inverse association between age and AKI severity in this cohort is biologically plausible. Younger children and infants have lower renal reserve, greater susceptibility to volume depletion, and immature tubular concentrating capacity, which may make them more vulnerable to ischemic and nephrotoxic insults in the PICU [23]. Reduced renal blood-flow autoregulation and greater sensitivity to vasoconstrictive mediators during sepsis and shock may also contribute to greater AKI severity in younger patients. These findings support heightened AKI vigilance and early fluid management strategies in infants and young children admitted to the PICU.

Clinical trajectory worsened substantially with advancing AKI stage. The high rate of inotropic support among Stage 3 patients (44.4%) illustrates the bidirectional relationship between cardiac dysfunction and renal impairment, often described as cardiorenal syndrome. Despite this severity, RRT was used in only 4.3% of the cohort and 11.1% of Stage 3 patients. This rate is lower than the 20–30% reported in some high-income settings [24]. Low RRT use may reflect resource limitations, higher thresholds for nephrology consultation (which occurred in 30.2% of cases), conservative clinical decision-making, or successful medical management of fluid overload and electrolyte disturbances in selected patients. Given the high mortality in Stage 3 AKI, this finding raises the hypothesis that standardized nephrology referral protocols and clearer institutional RRT initiation thresholds could influence outcomes. Prospective studies with granular fluid-balance and decision-audit data are needed to evaluate this possibility. Studies incorporating validated RRT initiation thresholds, such as fluid overload greater than 10–15% of body weight [25], may be especially informative.

The relatively conservative approach to RRT initiation in this cohort deserves further study. Emerging evidence supports earlier continuous RRT (CRRT) in selected pediatric patients with severe AKI and fluid overload [14,26], even in the absence of absolute biochemical indications. Standardized fluid overload monitoring and clear institutional RRT initiation protocols, developed in collaboration with pediatric nephrology, may represent a meaningful quality-improvement opportunity.

Overall PICU mortality in this AKI cohort was 16.7%, comparable to rates reported in developed countries (10–20%) [8,24], but lower than the 30–40% reported in some MENA studies [21,27]. The exclusion of neonates and patients with DNR status, who have higher baseline mortality risk, likely contributed to the lower overall mortality rate. However, mortality increased to 27.8% in Stage 3 AKI, supporting the prognostic value of KDIGO staging. In the multivariable model, inotrope requirement, number of complications, and PRISM IV score were associated with mortality, whereas AKI stage itself was not independently significant after adjustment. This pattern suggests that AKI often occurs as part of broader multiorgan dysfunction against a background of severe baseline illness. The number of complications should be interpreted cautiously because some included events, such as fluid overload, metabolic acidosis, hyperkalemia, and pulmonary edema, may be consequences or severity markers of AKI. Therefore, this finding should not be interpreted as evidence of a causal pathway independent of AKI severity. Clinically, the results support early hemodynamic stabilization, prevention of secondary organ failure, and timely kidney-focused follow-up as key priorities [28,29].

This study has several strengths. The use of standardized KDIGO criteria improves comparability with international literature, and the five-year dataset provides useful regional information on pediatric AKI in a tertiary PICU. The strict exclusion criteria also helped isolate AKI associated with acute critical illness. However, several limitations should be acknowledged. First, the retrospective, single-center design limits generalizability. Second, reliance on estimated baseline GFR for patients without prior creatinine measurements may have caused AKI stage misclassification in some patients. Third, although admission urine output was available for all 162 AKI patients in the analysis dataset, the dataset did not contain duration-specific UOP staging fields. Therefore, the final KDIGO stage could not be decomposed into creatinine-only, urine-output-only, and combined-criteria categories. Similarly, although initial and highest creatinine values were available for all 162 AKI patients and GFR values were available for 160 patients, the dataset did not include a separate variable indicating whether baseline creatinine was measured from prior records or estimated using the Schwartz formula. These limitations may affect the accuracy of AKI staging and may reduce direct comparability with studies that report criterion-specific staging or measured versus estimated baseline creatinine. Fourth, long-term follow-up data were not available to assess renal recovery, AKD, or progression to CKD. Fifth, any changes in PICU practice, nephrology consultation availability, or RRT initiation protocols after December 2022 were not captured. Finally, detailed nephrotoxin exposure and serial fluid-balance data were not analyzed, although nephrotoxic medication exposure is an important and modifiable contributor to pediatric AKI [30], and these factors may have influenced AKI staging and outcomes. The mortality model should also be interpreted cautiously because it included four variables with only 27 deaths, giving an EPV of 6.75. Although simultaneous entry was used to avoid stepwise selection bias, the estimates may be unstable and should be confirmed in larger prospective cohorts.

## 5. Conclusions

AKI was a clinically important complication among critically ill children, affecting approximately one in seven eligible PICU admissions in this cohort. Increasing KDIGO severity was consistently associated with adverse outcomes, including prolonged mechanical ventilation, longer PICU stay, greater morbidity, and higher mortality. Younger age and a greater burden of clinical complications were associated with AKI severity, while hemodynamic instability requiring inotropic support showed the strongest association with mortality in the multivariable model.

The low use of RRT despite substantial Stage 3 AKI raises the hypothesis that protocol standardization and earlier nephrology involvement could improve outcomes, a question that should be addressed in prospective studies with detailed fluid-balance and decision-audit data. Future multicenter studies incorporating fluid-balance metrics, nephrotoxin exposure, biomarkers, AKI-to-AKD transition assessment, and long-term renal follow-up are needed to refine risk stratification and guide timely intervention in this vulnerable population.

## Figures and Tables

**Table 1:** Demographic characteristics of the study cohort by AKI stage.

Variable	Stage 1 (n = 44)	Stage 2 (n = 64)	Stage 3 (n = 54)	Total (n = 162)	*p*
Age_years, Median (IQR)	7.0 (2.8–10.0)	3.0 (0.6–9.0)	1.0 (0.4–4.0)	3.0 (0.7–9.0)	<0.001
Sex (Male), n (%)	24 (54.5)	33 (51.6)	29 (53.7)	86 (53.1)	0.949
Weight kg, Median (IQR)	20.5 (11.5–34.2)	13.2 (6.8–24.0)	7.8 (4.2–15.0)	12.2 (6.0–24.8)	<0.001
Height cm, Median (IQR)	117.0 (93.0–136.5)	93.0 (64.5–127.5)	65.5 (55.5–99.0)	91.5 (64.0–130.0)	<0.001
Comorbidities present, n (%)	26 (59.1)	43 (67.2)	28 (51.9)	97 (59.9)	0.237
PRISM IV Score, Median (IQR)	8.5 (2.8–13.5)	7.0 (4.0–15.2)	10.0 (6.2–16.8)	9.0 (4.2–16.0)	0.204
Primary Reason (Medical), n (%)	27 (61.4)	47 (73.4)	53 (98.1)	127 (78.4)	<0.001

**Table 2:** Ordinal logistic regression analysis of predictors for acute kidney injury severity.

						95% Confidence Interval	
Predictor	Estimate	SE	Z	*p*	Odds Ratio	Lower	Upper
Age in years	−0.1244	0.056	−2.24	0.03	0.883	0.789	0.983
Gender:							
Male—Female	−0.1121	0.409	−0.27	0.78	0.894	0.399	1.998
Any underlying conditions & comorbidities:							
Yes—No	0.0918	0.425	0.216	0.83	1.096	0.477	2.538
Did the patient require inotropes:							
Yes—No	0.8817	0.457	1.928	0.05	2.415	0.99	6.00
Duration of mechanical ventilation	−0.0886	0.071	−1.25	0.21	0.915	0.792	1.049
Num of Comp	0.4406	0.219	2.015	0.04	1.554	1.019	2.417
Primary Reason Binary:							
Surgical—Medical	−1.2241	0.507	−2.42	0.02	0.294	0.107	0.786
Length of PICU stay days	0.0845	0.052	1.618	0.11	1.088	0.987	1.214

**Table 3:** PICU mortality, unadjusted and multivariable logistic regression results.

Predictor	Comparison	Unadjusted OR	95% CI	*p*	Adjusted OR	95% CI	*p*
Gender	Female, Male	1.35	0.58, 3.12	0.482	2.24	0.63, 7.92	0.212
Require Inotropes	No, Yes	17.04	6.19, 46.90	<0.001	8.51	2.44, 29.63	<0.001
Number of Complications	1.0, 6.0	4.73	2.75, 8.12	<0.001	4.38	2.28, 8.42	<0.001
PRISM IV	0.0, 43.0	1.05	1.01, 1.09	0.018	1.07	1.00, 1.14	0.042

Note. Unadjusted models contain one predictor each; the multivariable model includes all predictors simultaneously. OR = odds ratio. CI = 95% confidence interval. Statistical significance was set at *p* < 0.05. Sample size was n = 162. Positive outcome coded as 1: Not Survived. Reference categories are as follows. Gender: Female. Require Inotropes: No.

## Data Availability

The de-identified datasets generated and analyzed during the current study are available from the corresponding author upon reasonable request, subject to approval by the Institutional Review Board of King Abdulaziz University Hospital and in accordance with institutional data governance policies.

## Abbreviations

The following abbreviations are used in this manuscript:

AKI	Acute Kidney Injury
PICU	Pediatric Intensive Care Unit
KDIGO	Kidney Disease: Improving Global Outcomes
CKD	Chronic Kidney Disease
RRT	Renal Replacement Therapy
GFR	Glomerular Filtration Rate
UOP	Urine Output
PRISM IV	Pediatric Risk of Mortality Score (version IV)
VFDs	Ventilator-Free Days
DNR	Do-Not-Resuscitate
MODS	Multiorgan Dysfunction Syndrome
MENA	Middle East and North Africa
AWARE	Assessment of Worldwide Acute Kidney Epidemiology
SD	Standard Deviation
IQR	Interquartile Range
ANOVA	Analysis of Variance
OR	Odds Ratio
CI	Confidence Interval
ESRD	End-Stage Renal Disease
HFNC	High-Flow Nasal Cannula
NIV	Non-Invasive Ventilation
CPAP	Continuous Positive Airway Pressure
BiPAP	Bilevel Positive Airway Pressure
